# Supplementary material for: “What’s This Bug?” Questions from the Public Provide Relevant Information on Species Distribution and Human–Insect Interactions
Source: Insects. 2021 Oct 9;12(10):921. doi: 10.3390/insects12100921 (PMC8540344; doi:10.3390/insects12100921)
Supplement: Supplementary file 1 [file insects-12-00921-s001.zip › insects-1414956-SI.pdf]

Supplementary material

Table S1. Requests received in 2010, 2011, 2017 and 2018, per country.

Country	Number of requests
Canada	3085
France	33
United-States	20
Mexico	14
Brazil	6
Costa Rica	4
Italy	4
Australia	3
Belgium	3
China	3
Cuba	3
Afghanistan	2
Algeria	2
Cambodia	2
Cameroon	2
Nicaragua	2
Panama	2
Dominican Republic	2
Thaïland	2
South Africa	1
Angola	1
Argentina	1
Austria	1
Georgia	1
Haiti	1
Indonesia	1
New Caledonia	1
Peru	1

Democratic Republic of Congo	1
Rwanda	1
Switzerland	1
Tahiti	1
Togo	1
Uruguay	1
Venezuela	1
Unknown	953
